# Comparison Between Treadmill and Bicycle Ergometer Exercises in Terms of Safety of Cardiopulmonary Exercise Testing in Patients With Coronary Heart Disease

**DOI:** 10.3389/fcvm.2022.864637

**Published:** 2022-06-20

**Authors:** Chuan Ren, Jingxian Zhu, Tao Shen, Yanxin Song, Liyuan Tao, Shunlin Xu, Wei Zhao, Wei Gao

**Affiliations:** ^1^Department of Cardiology and Institute of Vascular Medicine, Peking University Third Hospital, National Health Commission, Key Laboratory of Cardiovascular Molecular Biology and Regulatory Peptides, Key Laboratory of Molecular Cardiovascular Science of Ministry of Education, Beijing Key Laboratory of Cardiovascular Receptors Research, Beijing, China; ^2^Department of Sports Medicine, Peking University Third Hospital, Institute of Sports Medicine, Peking University, Beijing, China; ^3^Research Center of Clinical Epidemiology, Peking University Third Hospital, Beijing, China; ^4^Physical Examination Center of Peking University Third Hospital, Beijing, China

**Keywords:** cardiopulmonary exercise testing, safety, stimulation mode, treadmill, bicycle ergometer, coronary heart disease

## Abstract

**Background:**

Cardiopulmonary exercise testing (CPET) is used widely in the diagnosis, exercise therapy, and prognosis evaluation of patients with coronary heart disease (CHD). The current guideline for CPET does not provide any specific recommendations for cardiovascular (CV) safety on exercise stimulation mode, including bicycle ergometer, treadmill, and total body workout equipment.

**Objective:**

The aim of this study was to explore the effects of different exercise stimulation modes on the occurrence of safety events during CPET in patients with CHD.

**Methods:**

A total of 10,538 CPETs, including 5,674 performed using treadmill exercise and 4,864 performed using bicycle ergometer exercise at Peking University Third Hospital, were analyzed retrospectively. The incidences of CV events and serious adverse events during CPET were compared between the two exercise groups.

**Results:**

Cardiovascular events in enrolled patients occurred during 355 CPETs (3.4%), including 2 cases of adverse events (0.019%), both in the treadmill group. The incidences of overall events [235 (4.1%) vs. 120 (2.5%), *P* < 0.001], premature ventricular contractions (PVCs) [121 (2.1%) vs. 63 (1.3%), *P* = 0.001], angina pectoris [45 (0.8%) vs. 5 (0.1%), *P* < 0.001], and ventricular tachycardia (VT) [32 (0.6%) vs. 14 (0.3%), *P* = 0.032] were significantly higher in the treadmill group compared with the bicycle ergometer group. No significant difference was observed in the incidence of bradyarrhythmia and atrial arrhythmia between the two groups. Logistic regression analysis showed that the occurrence of overall CV events (*P* < 0.001), PVCs (*P* = 0.007), angina pectoris (*P* < 0.001), and VT (*P* = 0.008) was independently associated with the stimulation method of treadmill exercise. In male subjects, the occurrence of overall CV events, PVCs, angina pectoris, and VT were independently associated with treadmill exercise, while only the overall CV events and angina pectoris were independently associated with treadmill exercise in female subjects.

**Conclusion:**

In comparison with treadmill exercise, bicycle ergometer exercise appears to be a safer exercise stimulation mode for CPET in patients with CHD.

## Introduction

Cardiopulmonary exercise testing (CPET) is a comprehensive and practical examination of individuals conducted to investigate the interaction among the heart, lung, muscular, and circulatory system and their reserve capacity during incremental-load exercise. CPET is used widely in the diagnosis, exercise therapy, and prognosis evaluation of patients with coronary heart disease (CHD). CPET is also commonly used in other heart-related conditions, such as heart failure, cardiomyopathy, and pulmonary hypertension (1). In certain athletes undergoing exercise testing, other forms of diagnoses that are potentially characterized by malignant arrhythmias during effort, such as channelopathies, particularly catecholaminergic polymorphic ventricular tachycardia (2) and long QT syndrome (3), should be considered. As the application of CPET is becoming further widespread, increasing attention is being paid to the safety concerns associated with this exercise testing method. The stimulation modes used commonly in exercise testing include bicycle ergometers, treadmills, total body workout equipment, etc., among which the first two are the most frequently used ones. The current guideline for CPET does not provide any specific recommendations on which of the exercise stimulation modes to use for better results. According to a previous scientific statement issued by the American Heart Association, the incidences of fatal adverse events and events requiring medical intervention during exercise testing are estimated to be <0.01 and <0.2, respectively (4). However, no large-scale research has been conducted so far on the effect of different exercise stimulation modes on the safety of CPET. Therefore, the present study aimed to review the data on patients with CHD who underwent CPET based on treadmill or lower-body bicycle ergometer exercise. The objective was to explore the effect of different exercise stimulation modes on the occurrence of safety events during CPET in these patients.

## Materials and Methods

### Subjects

Patients diagnosed with CHD who underwent CPET at Peking University Third Hospital between July 2006 and September 2019 were enrolled in the present retrospective study. The inclusion criteria were as follows: age > 18 years, diagnosis of CHD, and having undergone CPET. Patients with incomplete information were excluded from this study. The study protocol was approved by the Ethics Committee of Peking University Third Hospital [2019 No. 266–01]. Informed consent was obtained from all included subjects.

### Data Collection

The collected data included demographic characteristics (name, gender, date of birth, and body mass index), history of hypertension, hyperlipidemia, diabetes, smoking, and exercise habit, and the current medication for cardiovascular (CV) disease.

### Cardiopulmonary Fitness Testing

The CPET system (Medical Graphics Corporation, St Louis, United States) was adopted for testing. The treadmill exercise was conducted following the Bruce protocol (5). The bicycle ergometer exercise was conducted following the Ramp protocol (6). Patients were encouraged to perform the symptom-limited exercise, with self-reported fatigue scores between 17 and 20 (7) and respiratory exchange rate (RER) ≥ 1.1. The exercise was terminated if any of the following occurred: typical angina pectoris; evident symptoms and signs; dyspnea, paleness, cyanosis, dizziness, vertigo, unsteady gait, ataxia, ischemic claudication; increased discomfort or pain in the lower body during the exercise; horizontal or down-sloping ST-segment depression with at least 0.1 mV or damaged ST-segment elevation with at least 2.0 mV; any of the malignant arrhythmias, such as ventricular tachycardia (VT), ventricular fibrillation, frequent or R-on-T premature ventricular contractions (PVCs), sustained supraventricular tachycardia, atrial flutter/fibrillation, etc.; systolic blood pressure (SBP) remaining constant or decreasing by at least 10 mmHg during the exercise; hyperpiesia with SBP over 220 mmHg; intraventricular block during the exercise; and requests from patients to terminate the exercise. All data generated in this study were interpreted by experienced cardiologists. The criteria for positive exercise stress test were as follows: horizontal or down-sloping ST-segment depression, 60–80 ms after the J point, with at least 0.1 mV for at least 2 min in the leads with a dominant R wave; up-sloping ST-segment depression with at least 0.2 mV accompanied by ST-segment elevation in lead aVR with at least 0.1 mV; arched ST-segment elevation with at least 0.1 mV and for at least 1 min in lead with a non-pathological Q wave. The indicators measured during CPET included peak SBP (SBPpeak), peak heart rate (HRpeak), peak oxygen uptake (VO_2_peak), oxygen uptake at the anaerobic threshold (VO_2_@AT), peak oxygen pulse (VO_2_/HR), and peak RER (RERpeak). The anaerobic threshold was determined using the V-slope method.

### Definition of Cardiovascular Events and Serious Adverse Events

According to the objective of the present study, investigators reviewed the CPET data of all included patients, which contained the records of all the events that occurred during CPET. A CV event was defined as the event with any of the following conditions (6): (1) PVCs, including new-onset frequent PVCs (over 5 beats per minute) or increased PVCs during exercise or recovery; (2) atrial arrhythmia, referring to the new-onset frequent premature atrial contractions (over 5 beats per minute) or increased premature atrial contractions, atrial tachycardia (AT), atrial flutter, atrial fibrillation, and paroxysmal supraventricular tachycardia during exercise or recovery; (3) angina pectoris, which referred to typical ischemic chest pain accompanied by ischemia-like ECG changes; (4) sustained or non-sustained VT; and (5) bradyarrhythmia, including new-onset sinus arrest, atrioventricular block, left/right bundle branch block, and aberrant ventricular conduction during the examination. A serious adverse event was defined as the event with any of the following conditions (8): (1) death within 48 h of CPET; (2) acute myocardial infarction within 24 h of CPET; (3) sustained VT (duration > 30 s); (4) requirement of external defibrillation or implantation of a subcutaneous implantable cardioverter-defibrillator; (5) syncope; (6) requirement of advanced life support; and (7) requirement of hospitalization or emergency treatment due to incidents that occurred during CPET.

### Statistical Analysis

SPSS 20.0 was employed for statistical analysis. Quantitative data with normal distribution were presented as mean ± standard deviation (mean ± SD), while the non-normal data were presented as median (interquartile range) [median (IQR)]. The data of the treadmill group and bicycle ergometer group were compared using an independent-samples *t*-test for normally distributed data and the Mann–Whitney *U* test for non-normal data. Categorical variables were compared using the chi-square test. Logistic regression analysis was performed to explore the correlation between different types of exercises and the occurrence of CV events. *P* < 0.05 was considered the threshold of statistical significance.

## Results

### Basic Characteristics

A total of 10,538 CPETs conducted with 8,975 patients were included in the present study. The CPETs included 5,674 performed using treadmill exercise and 4,864 performed using bicycle ergometer exercise. The mean age in the treadmill exercise group was 59.5 ± 10.6 years, and there were 4,650 men (82.0%). The mean age in the bicycle ergometer exercise group was 61.1 ± 10.5 years old, and there were 3,808 men (78.3%). As shown in Table 1, the proportions of male patients, smokers, those having the habit of exercising, and the subjects consuming β-blockers, statins, angiotensin-converting enzyme inhibitors/angiotensin receptor blocker (ACEI/ARB), nitrates, calcium antagonists, and antiplatelet drugs were significantly higher in the treadmill group (all *P* < 0.05) compared with the bicycle ergometer exercise group. The mean age and the proportions of subjects with hyperlipidemia were significantly lower in the treadmill group compared with the bicycle ergometer group (all *P* < 0.05).

**TABLE 1:** Comparison of baseline characteristics between the treadmill group and the bicycle ergometer group.

	Treadmill (*n* = 5674)	Bicycle ergometer (*n* = 4864)	*P*
**Demographics**			
Age	59.5 ± 10.6	61.1 ± 10.5	<0.001
Male, n(%)	4650 (81.9)	6638 (63.4)	<0.001
BMI, ≥ 23.9 kg/m^2^, n(%)	4071 (71.7)	3489 (71.7)	0.387
Smoker, n(%)	3373 (59.4)	2289(47.1)	<0.001
Having exercise habits	3980 (70.1)	3267 (67.2)	0.023
**Comorbidity**			
Hypertension, n(%)	3740 (61.2)	3022 (62.1)	0.306
Hyperlipidemia, n(%)	3400(60.0)	3206 (65.9)	<0.001
Diabetes, n(%)	1725 (30.4)	1475 (30.3)	0.932
**Medications**			
β-blockers, n(%)	3753(66.1)	2546 (52.3)	<0.001
Statins, (%)	4962 (87.5)	3926 (80.7)	<0.001
ACEI/ARB, n(%)	2560(45.1)	1437 (29.5)	<0.001
Calcium antagonists, (%)	1169 (20.6)	923 (19.0)	0.037
Antiplatelet drugs, n(%)	5188 (91.4)	4166 (85.6)	<0.001
Long-acting nitrates, n(%)	1095 (19.3)	798 (16.4)	<0.001
Diuretics, n(%)	199 (3.5)	168 (3.5)	0.882

*BMI, body mass index; ACEI/ARB, angiotensin-converting enzyme inhibitors/angiotensin receptor blocker.*

### Cardiopulmonary Exercise Testing Results

The median RERpeak of all subjects was at 1.11 (1.04, 1.19), suggesting a good level of exertion during CPET. As shown in Table 2, the proportion of positive exercise stress was significantly higher in the treadmill group compared with the bicycle ergometer group (*P* < 0.001). The VO_2_peak, VO_2_@AT, HRpeak, and peak SBP*HR of the treadmill group were significantly higher than the corresponding values in the bicycle ergometer group, while the RERpeak of the treadmill group was significantly lower than that of the bicycle ergometer group (all *P* < 0.001).

**TABLE 2:** Comparison of the CPET results between the treadmill group and the bicycle ergometer group.

	Treadmill (*n* = 5674)	Bicycle ergometer (*n* = 4864)	*P*
Positive exercise stress test,%	942 (16.4)	410 (8.4)	<0.001
VO_2_peak, mL/kg/min	23.6 (20.0, 27.0)	18.3 (15.0, 21)	<0.001
VO_2_@AT, mL/kg/min	19.2 (16.3, 22.7)	11.6 (9.9, 13.6)	<0.001
HRpeak, bpm	136 (125, 147)	125 (111, 138)	<0.001
SBPpeak, mmHg	169 (152, 187)	171 (151, 189)	0.055
Peak SBP*HR, mmHg*bpm	22965 (19674, 26410)	20944 (17264, 25128)	<0.001
RERpeak	1.08 (1.02, 1.15)	1.16 (1.09, 1.24)	<0.001

*VO_2_ peak, peak oxygen uptake; VO_2_@AT, oxygen uptake at the anaerobic threshold; HRpeak, peak heart rate; SBPpeak, peak systolic blood pressure; RERpeak, peak respiratory exchange ratio; CPET, cardiopulmonary exercise testing.*

### Comparison of Overall Events and Serious Adverse Events Between the Treadmill Group and Bicycle Ergometer Group

**FIGURE 1:**
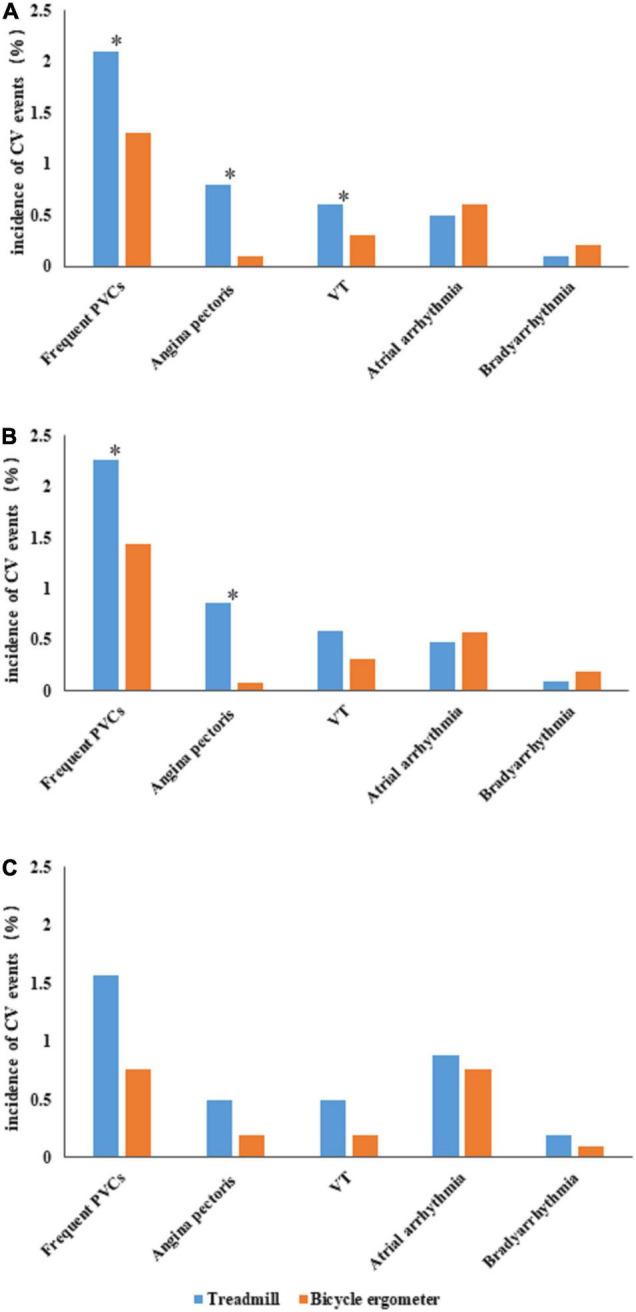
Comparison of cardiovascular (CV) events between the treadmill group and bicycle ergometer group. **(A)** Depicts the incidences of various CV events in the two groups. **(B)** Depicts the incidences of various CV events in the male subjects of the two groups. **(C)** Depicts the incidences of various CV events in the female subjects of the two groups. The “*” symbol indicates a significant difference in the incidence of CV events between the two groups (*P* < 0.05). CV, cardiovascular; PVCs, premature ventricular contractions; VT, ventricular tachycardia.

Cardiovascular events occurred during a total of 355 CPETs (3.4%) in the enrolled patients, including 2 male patients with serious adverse events (0.019%). Both patients with serious adverse events were hospitalized after sustained ventricular tachycardia during CPET performed using treadmill exercise. As depicted in Figure 1A, the incidences of overall events [235 (4.1%) vs. 120 (2.5%), *P* < 0.001], PVCs [121 (2.1%) vs. 63 (1.3%), *P* = 0.001], angina pectoris [45 (0.8%) vs. 5 (0.1%), *P* < 0.001], and VT [32 (0.6%) vs. 14 (0.3%), *P* = 0.032] were significantly higher in the treadmill group compared with the bicycle ergometer group. No significant differences were observed in the incidences of bradyarrhythmia and atrial arrhythmia between the two groups.

The logistic regression analysis was performed using the occurrence of CV events as the dependent variable and adjusting for gender, age, smoking, exercise habits, previous comorbidities, current medication, positive exercise stress test, and VO_2_peak. The results suggested that the occurrence of overall CV events was independently associated with the exercise stimulation mode in the treadmill exercise group [0.503 (95% *CI* 0.388–0.653), *P* < 0.001]. The subsequent logistic regression analysis performed with the occurrence of various types of CV events as the dependent variable and adjusting for the above covariates revealed that the occurrences of PVCs [0.611 (95% *CI* 0.427–0.876), *P* = 0.007), angina pectoris [0.063 (95% *CI* 0.024–0.167), *P* < 0.001], and VT [0.378 (95% *CI* 0.184–0.777), *P* = 0.008] were independently associated with treadmill exercise, while bradyarrhythmia and atrial arrhythmia were not associated with the exercise stimulation modes (Table 3).

**TABLE 3:** Correlation analysis between the occurrence of CV events and treadmill exercise in all subjects using the logistic regression analysis.

	OR (95% CI)	*P*
Overall events	0.503 (0.388–0.653)	<0.001
Frequent PVCs	0.611 (0.427–0.876)	0.007
Angina pectoris	0.063 (0.024–0.167)	<0.001
VT	0.378 (0.184–0.777)	0.008
Atrial arrhythmia	1.183 (0.647–2.160)	0.586
Bradyarrhythmia	1.138 (0.322–4.020)	0.840

*PVCs, premature ventricular contractions; VT, ventricular tachycardia; CV, cardiovascular.*

### Association Between Exercise Stimulation Modes and the Occurrence of Cardiovascular Events Mediated by Gender

#### Comparison of Cardiovascular Events in Male Subjects Between the Treadmill Group and Bicycle Ergometer Group

A total of 8,458 CPETs were conducted in male subjects, including 4,650 in the treadmill group and 3,808 in the bicycle ergometer group. As presented in Supplementary Table 1, the proportions of smokers and the subjects consuming β-blockers, statins, ACEI/ARB, nitrates, and antiplatelet drugs were significantly higher in the treadmill group compared with the bicycle ergometer group (all *P* < 0.05). The mean age and the proportion of subjects with hyperlipidemia were significantly lower in the treadmill group (all *P* < 0.05) compared with the bicycle ergometer group.

Furthermore, as presented in Supplementary Table 2, the proportion of positive exercise stress test, VO_2_peak, VO_2_@AT, HRpeak, and peak SBP*HR were significantly higher in the treadmill group compared with the bicycle ergometer group, while SBPpeak and RERpeak were significantly lower in the treadmill group (all *P* < 0.001).

The incidences of overall events [198 (4.3%) vs. 99 (2.6%), *P* < 0.001], PVCs [105 (2.3%) vs. 55 (1.4%), *P* = 0.006] and angina pectoris [40 (0.9%) vs. 3(0.1%), *P* < 0.001] were significantly higher in the treadmill group compared with the bicycle ergometer group. No significant differences were observed in the incidences of VT, bradyarrhythmia, and atrial arrhythmia between the two groups (Figure 1B).

When the logistic regression analysis was performed with the occurrences of overall events and different types of CV events as dependent variables and adjusting for age, smoking, exercise habits, previous comorbidities, current medication, positive exercise stress test, and VO_2_peak, it was revealed that the occurrences of overall CV events, PVCs, angina pectoris, and VT were independently associated with the exercise stimulation mode in the treadmill exercise group (Table 4).

**TABLE 4:** Correlation analysis between the occurrence of CV events and treadmill exercise in the male subjects using the logistic regression analysis.

	OR (95% CI)	*P*
Overall events	0.509 (0.383–0.677)	<0.001
Frequent PVCs	0.643 (0.438–0.944)	0.024
Angina pectoris	0.045 (0.013–0.550)	<0.001
VT	0.374 (0.172–0.813)	0.013
Atrial arrhythmia	1.221 (0.603–2.472)	0.578
Bradyarrhythmia	1.915 (0.457–8.031)	0.374

*PVCs, premature ventricular contractions; VT, ventricular tachycardia; CV, cardiovascular.*

#### Comparison of Cardiovascular Events in Female Subjects Between the Treadmill Group and Bicycle Ergometer Group

A total of 2,080 CPETs were conducted with female subjects, including 1,024 in the treadmill group and 1,056 in the bicycle ergometer group. As presented in Supplementary Table 3, the proportions of smokers and those having a habit of exercising were significantly higher in the treadmill group compared with the bicycle ergometer group (all *P* < 0.05). The proportions of subjects consuming β-blockers, statins, ACEI/ARB, calcium antagonists, antiplatelet drugs, and nitrates were also significantly higher in the treadmill group (all *P* < 0.05), while the mean age was significantly lower in the treadmill group (*P* < 0.05). Furthermore, as presented in Supplementary Table 4, the proportions of positive exercise stress test, VO_2_peak, VO_2_@AT, HRpeak, SBPpeak, and peak SBP*HR were significantly higher in the treadmill group compared with the bicycle ergometer group, while RERpeak was significantly lower in the treadmill group (all *P* < 0.001).

In female subjects, the incidences of overall events [37 (3.6%) vs. 21 (2.0%), *P* < 0.001] were significantly higher in the treadmill group compared with the bicycle ergometer group. No significant difference was observed in the incidences of PVCs, angina pectoris, VT, bradyarrhythmia, and atrial arrhythmia between the two groups (Figure 1C).

The logistic regression analysis performed with the occurrences of overall events and different types of CV events as dependent variables and adjusting for age, smoking, exercise habits, previous comorbidities, current medication, positive exercise stress test, and VO_2_peak revealed that the occurrences of overall CV events and angina pectoris were independently associated with the exercise stimulation mode in the treadmill exercise group (Table 5).

**TABLE 5:** Correlation analysis between the occurrence of CV events and treadmill exercise in the female subjects using the logistic regression analysis.

	OR (95% CI)	*P*
Overall events	00.424 (0.217–0.827)	0.012
Frequent PVCs	0.428 (0.152–1.206)	0.108
Angina pectoris	0.067 (0.009–0.511)	0.009
VT	0.294 (0.0.35–2.478)	0.260
Atrial arrhythmia	1.071 (0.312–3.681)	0.913
Bradyarrhythmia	0.165 (0.008–3.431)	0.245

*PVCs, premature ventricular contractions; VT, ventricular tachycardia; CV, cardiovascular.*

## Discussion

In the present study, the CPET data of patients with CHD were reviewed to compare the incidences of CV events between different exercise stimulation modes. It was revealed that the probability of occurrence of CV events in patients with CHD was higher in the treadmill group compared with the bicycle ergometer group. Specifically, the occurrences of PVCs, angina pectoris, and VT were independently associated with the stimulation mode of treadmill exercise.

The safety of the exercise stress test has remained a concern for a long time and has, therefore, been investigated in several studies. In contrast to traditional exercise stress tests that target heart rate as the endpoint, CPET recommends symptom-limited exercise stress tests that are not limited to reaching a target heart rate. The present study revealed that the overall incidence of CV events in patients with CHD was 3.4%, with the incidence of serious adverse events being only 0.019%. This is consistent with the findings of previous studies performed on CV disease (9). For instance, the HF-ACTION trial evaluated the safety of CPET in 2,037 patients with heart failure (with a median left ventricular ejection fraction of 25%), in which only 2 cases of ventricular arrhythmias were observed while no fatal events occurred (10). A study conducted with subjects at high risk of CV diseases (including CHD, valvular heart disease, heart failure, and congenital heart disease) reported a similar percentage incidence of serious adverse events during CPET (0.16%) (8). Therefore, it is generally considered that symptom-limited exercise tests are relatively safe regardless of the exercise stimulation modes.

The stimulation modes commonly used in exercise tests include upper or lower body bicycle ergometer, treadmill, and total body workout equipment, among which lower body bicycle ergometer and treadmill are the most frequently used ones. Bicycle ergometer offers the advantages of smaller space occupation, less noise, convenient quantification of workload, less interference in ECG, and avoidance of physical injury that could have been caused due to unstable standing (4). The current guidelines for CPET present no significant preference for a particular type of exercise stimulation mode, and the exercise selection relies mainly on local conditions and patient cooperation during the exercise. For instance, the probability of opting for a bicycle ergometer is higher among patients with obesity, orthopedic diseases, and nervous system disorders. In terms of exercise safety, the present study reveals no significant association between the occurrence of serious adverse events and the exercise stimulation modes. However, a bicycle ergometer remained the safer choice considering the CV events, including angina pectoris and various arrhythmias. The mechanism behind this phenomenon is worth exploring. First, in maximal exercise, treadmill exercise induces greater stimulation to the heart and the lung compared with bicycle ergometers. Previous studies have demonstrated that, in the exercise tests using a bicycle ergometer, untrained subjects usually request to terminate the test due to quadriceps femoris muscle fatigue, thereby achieving 5%–20% lower VO_2_peak on average compared with that obtained using treadmill exercise (4, 11). A similar result was observed in the present study as well. The VO_2_peak in maximal exercise depends on the peak cardiac output and arteriovenous oxygen difference, while the difference between the arterial and venous oxygen partial pressures in maximal exercise under the two exercise modes is not significant. The data provided by Hermansen et al. suggest that the influence of stroke volume on cardiac output exerts a significant impact on VO_2_peak during cycling and running and that stroke volume under the stimulation of treadmill exercise is larger than that with bicycle ergometers (12, 13). Related studies have demonstrated that the HRpeak of subjects performing treadmill exercise is 10–25 times higher than those performing the bicycle ergometer exercise, which is similar to the findings of the present study. The peak SBP*HR was also higher in the treadmill group, indicating higher myocardial oxygen consumption. Moreover, even at the same exercise intensity, HR and plasma lactate concentrations were higher in the subjects performing treadmill exercise compared with those performing the bicycle ergometer exercise (14, 15). But the multivariate regression analysis in the present study revealed that the correlation between exercise stimulation methods and events is independent of VO_2_peak. We think that the different probability of events caused by different stimulation methods may not only depend on higher heart rate or oxygen uptake but also depend on other factors, such as sympathetic excitability and postures. The regulation of the sympathetic nervous system varies under different exercise stimulation modes. Both treadmill and bicycle ergometer exercises are performed mainly using the muscle contraction of the lower body. The main muscle group involved in cycling is the quadriceps femoris, while plantar flexor muscle and quadriceps femoris play key roles during treadmill exercise. As the slope increases, the recruitment of the quadriceps femoris gradually increases (16). Studies have demonstrated that blood flow distribution in the muscles recruited during exercise is regulated by the sympathetic nervous system (17). Moreover, different postures also exert different effects on breathing-related muscles. The stimulation under treadmill and bicycle ergometer exercises produces different ventilatory responses, including differences in exercise-induced arterial hypoxemia, oxygen diffusion capacity, ventilation fatigue, and respiratory mechanics (18). This could also be the reason underlying the greater number of events under the stimulation of treadmill exercise. However, further studies are required to decipher the reason for the higher probability of occurrence of ventricular arrhythmia rather than atrial arrhythmia and bradyarrhythmia during treadmill exercise.

According to previous studies, CV events during exercise stress tests or the marathon sport appear to be more common in men (8, 19). In addition, differences have been reported in response to exercise, including VO_2_peak, between men and women (20). In non-athletes or patients with heart disease, the VO_2_peak of women is usually 5–15% lower than that of men undergoing equivalent training intensity and having similar age (21), and these observations were consistent in different age groups (22). The differences in the physical characteristics (such as body size, muscle mass, sex hormones, etc.) between men and women could also affect CV safety during exercise tests. Therefore, a subgroup analysis based on gender was conducted in the present study. The results revealed no significant difference in the occurrence of all CV events between individuals of different genders in both stimulation modes. However, the situation of a specific event was slightly different between different genders. On the other hand, the probability of occurrence of angina pectoris was higher on the treadmill for both men and women. The probability of occurrence of all kinds of arrhythmias was higher on the treadmill, which appeared to be further evident in males. The possible explanations for this difference in arrhythmias could be that women exhibit a lower intensity of the pressoreceptor response to exercise, which leads to a lesser increase in the sympathetic nervous system activity compared with men (23, 24). Changes in the ejection fraction from rest to maximal and submaximal exercise (25), the increase in the cardiac output, and SBP*HR (26) are also reported to be relatively lower in women. Notably, most of the female subjects included in the present study were menopausal women. Previous studies have suggested that the resting muscle sympathetic nerve activity of younger women is lower than that of men due to the influence of sex hormones (27, 28). However, with the accelerated increase in sympathetic nerve activity during menopause (29), the influence of sympathetic nerve activity on hemodynamics during exercise could be further significant in premenopausal women (30). Therefore, further studies are warranted to confirm whether the occurrence of CV events in men and women under different stimulation modes is influenced by sympathetic nervous system activity.

As with all studies, the present study also has certain limitations. It was conducted retrospectively with a single-center design due to which the causal relationship between the exercise stimulation modes and the events could not be determined. In addition, various confounding factors, such as myocardial infarction or heart failure, in the enrolled patients could have led to a certain degree of bias in the results obtained.

## Data Availability Statement

The raw data supporting the conclusions of this article will be made available by the authors, without undue reservation.

## Ethics Statement

The studies involving human participants were reviewed and approved by the Ethics Committee of Peking University Third Hospital. The patients/participants provided their written informed consent to participate in this study. Written informed consent was obtained from the individual(s) for the publication of any potentially identifiable images or data included in this article.

## Author Contributions

CR, JZ, and TS participated in the study design, analyzed the data, and drafted the manuscript. YS collected the data for this study. LT assisted in the statistical analysis. SX assisted in drafting the manuscript. WZ and WG contributed to the conception of the study and also participated in its design and coordination. All authors read and approved the final version of the manuscript and also agreed with the order of presentation of the authors.

## Conflict of Interest

The authors declare that the research was conducted in the absence of any commercial or financial relationships that could be construed as a potential conflict of interest.

## Publisher’s Note

All claims expressed in this article are solely those of the authors and do not necessarily represent those of their affiliated organizations, or those of the publisher, the editors and the reviewers. Any product that may be evaluated in this article, or claim that may be made by its manufacturer, is not guaranteed or endorsed by the publisher.
