# Supplementary material for: Comparison Between Treadmill and Bicycle Ergometer Exercises in Terms of Safety of Cardiopulmonary Exercise Testing in Patients With Coronary Heart Disease
Source: Front Cardiovasc Med. 2022 Jun 20;9:864637. doi: 10.3389/fcvm.2022.864637 (PMC9251120; doi:10.3389/fcvm.2022.864637)
Supplement: Supplementary file 1 [file Table_1.DOCX]

**Supplemental Table 1. Comparison of baseline characteristics of male subjects between the treadmill group and the bicycle ergometer group**

|  | Treadmill  (n=6225) | Bicycle ergometer  (n=6638) | *P* |
| --- | --- | --- | --- |
| **Demographics** |  |  |  |
| Age | 57.6±12.7 | 57.0±13.6 | 0.027 |
| BMI≥23.9kg/m^2^, n(%), | 4394 (70.6) | 4688 (70.6) | 0.245 |
| Smoker, n(%) | 4027 (64.7) | 3447 (51.9) | <0.001 |
| Having exercise habits | 4158 (66.8) | 4170 (62.8) | <0.001 |
| **Comorbidity** |  |  |  |
| Hypertension, n(%) | 3463 (55.6) | 3435 (51.7) | <0.001 |
| Hyperlipidemia, n(%) | 3250 (52.2) | 3349 (50.5) | 0.046 |
| Diabetes, n(%) | 1563 (25.1) | 1513 (22.8) | 0.002 |
| **Medications** |  |  |  |
| β-blockers, n(%) | 3404 (54.7) | 2437 (36.7) | <0.001 |
| Statins, (%) | 4423 (71.1) | 3659 (55.1) | <0.001 |
| ACEI/ARB, n(%) | 2417 (38.8) | 1572 (23.7) | <0.001 |
| Calcium antagonists, (%) | 1124 (18.1) | 1168 (17.6) | 0.495 |
| Antiplatelet drugs, n(%) | 4530 (72.8) | 3681 (55.5) | <0.001 |
| Long-acting nitrates, n(%) | 928 (14.9) | 711 (10.7) | <0.001 |
| Diuretics, n(%) | 229 (3.7) | 194 (2.9) | 0.016 |
| Digoxin, n(%) | 17 (0.3) | 6 (0.1) | 0.014 |
| **Major diagnoses** |  |  |  |
| Coronary heart disease, n(%) | 4650 (74.7) | 3808 (57.4) | <0.001 |
| Heart failure, n(%) | 28 (0.5) | 34 (0.5) | 0.610 |
| Arrhythmia, n(%) | 182 (2.9) | 12 (0.2) | 0.090 |
| Valvular heart disease, n(%) | 23 (0.4) | 14 (0.2) | 0.093 |
| Cardiomyopathy, n(%) | 22 (0.4) | 13 (0.2) | 0.086 |
| Congenital heart disease, n(%) | 5 (0.1) | 1 (0.1) | 0.087 |
| Preoperative evaluation, n(%) | 154 (2.5) | 49 (0.7) | <0.001 |
| Check-up for discomfort, n(%) | 1161 (18.7) | 2107 (31.7) | <0.001 |

Abbreviation: BMI, body mass index; ACEI/ARB: angiotension converting enzyme inhibitors/angiotensin receptor blocker

**Supplemental Table 2. Comparison of CPET results in male subjects between the treadmill group and the bicycle ergometer group**

|  | Treadmill  (n=6225) | Bicycle ergometer (n=6638) | *P* |
| --- | --- | --- | --- |
| Positive exercise stress test, % | 1310 (21.0) | 1220 (18.4) | <0.001 |
| VO_2_peak, ml/kg/min | 24.8 (21.2, 28.6) | 19.6 (16.5, 23.3) | <0.001 |
| VO_2_@AT, ml/kg/min | 20.2 (17.0, 23.9) | 12.3 (10.4, 14.7) | <0.001 |
| HRpeak, bpm | 140 (127, 152) | 132 (117, 148) | <0.001 |
| SBPpeak, mmHg | 170 (153, 189) | 174 (155, 194) | <0.001 |
| Peak SBP*HR, mmHg*bpm | 23864 (20250, 27540) | 22869 (18477, 27531) | <0.001 |
| RERpeak | 1.09 (1.03, 1.15) | 1.17 (1.10, 1.25) | <0.001 |

Abbreviation: VO_2_peak, peak oxygen uptake; VO_2_@AT, oxygen uptake at anaerobic threshold; HRpeak, peak heart rate; SBPpeak, peak systolic blood pressure; RERpeak: peak respiratory exchange ratio.

**Supplemental Table 3. Comparison of baseline characteristics of female subjects between the treadmill group and the bicycle ergometer group**

|  | Treadmill  (n=2100) | Bicycle ergometer  (n=3830) | *P* |
| --- | --- | --- | --- |
| **Demographics** |  |  |  |
| Age | 60.6±11.6 | 58.2±13.2 | <0.001 |
| BMI≥23.9 kg/m^2^, n(%), | 1242 (59.1) | 2128 (55.6) | 0.022 |
| Smoker, n(%) | 115 (5.5) | 114 (3.0) | <0.001 |
| Having exercise habits | 1272 (60.6) | 2058 (53.7) | <0.001 |
| **Comorbidity** |  |  |  |
| Hypertension, n(%) | 1212 (57.7) | 1690 (44.1) | <0.001 |
| Hyperlipidemia, n(%) | 1081 (51.5) | 1676 (43.8) | <0.001 |
| Diabetes, n(%) | 519 (24.7) | 760 (19.8) | <0.001 |
| **Medications** |  |  |  |
| β-blockers, n(%) | 873 (41.6) | 802 (20.9) | <0.001 |
| Statins, (%) | 1145 (54.5) | 1406 (36.7) | <0.001 |
| ACEI/ARB, n(%) | 640 (30.5) | 697 (18.2) | <0.001 |
| Calcium antagonists, (%) | 481 (22.9) | 674 (17.6) | <0.001 |
| Antiplatelet drugs, n(%) | 1146 (54.6) | 1116 (29.1) | <0.001 |
| Long-acting nitrates, n(%) | 306 (14.6) | 224 (5.8) | <0.001 |
| Diuretics, n(%) | 97 (4.6) | 112 (2.9) | 0.001 |
| Digoxin, n(%) | 7 (0.3) | 2 (0.1) | 0.008 |
| **Major diagnoses** |  |  |  |
| Coronary heart disease, n(%) | 1024 (48.8) | 1056 (27.6) | <0.001 |
| Heart failure, n(%) | 10 (0.5) | 12 (0.3) | 0.324 |
| Arrhythmia, n(%) | 125 (6.0) | 134 (3.5) | <0.001 |
| Valvular heart disease, n(%) | 32 (1.5) | 17 (0.44) | <0.001 |
| Cardiomyopathy, n(%) | 13 (0.7) | 7 (0.2) | 0.006 |
| Congenital heart disease, n(%) | 3 (0.1) | 7 (0.3) | 0.72 |
| Preoperative evaluation, n(%) | 135 (6.429) | 426 (11.1) | <0.001 |
| Check-up for discomfort, n(%) | 758 (36.1) | 2171 (56.7) | <0.001 |

Abbreviation: BMI, body mass index; ACEI/ARB: angiotension converting enzyme inhibitors/angiotensin receptor blocker

**Supplemental Table 4. Comparison of CPET results in female subjects between the treadmill group and the bicycle ergometer group**

|  | Treadmill  (n=2100) | Bicycle ergometer (n=3830) | *P* |
| --- | --- | --- | --- |
| Positive exercise stress test, % | 397 (18.9) | 555 (14.5) | <0.001 |
| VO_2_peak, ml/kg/min | 21.1 (18.2, 24.3) | 17.2 (14.4, 20.1) | <0.001 |
| VO_2_@AT, ml/kg/min | 17.8 (15.4, 20.1) | 11.5 (9.8, 13.5) | <0.001 |
| HRpeak, bpm | 141 (128, 154) | 134 (119, 148) | <0.001 |
| SBPpeak, mmHg | 170 (153, 189) | 167 (149, 185) | <0.001 |
| Peak SBP*HR, mmHg*bpm | 23993 (20751, 27360) | 22078 (18419, 25702) | <0.001 |
| RERpeak | 1.08 (1.01, 1.14) | 1.14 (1.08, 1.23) | <0.001 |

Abbreviation: VO_2_peak, peak oxygen uptake; VO_2_@AT, oxygen uptake at anaerobic threshold; HRpeak, peak heart rate; SBPpeak, peak systolic blood pressure; RERpeak: peak respiratory exchange ratio.
